# Unravelling nuclear size control

**DOI:** 10.1007/s00294-019-00999-3

**Published:** 2019-05-31

**Authors:** Helena Cantwell, Paul Nurse

**Affiliations:** 1 Cell Cycle Laboratory, The Francis Crick Institute, London, NW1 1AT UK; 2 Laboratory of Yeast Genetics and Cell Biology, Rockefeller University, New York, NY 10065 USA; 3 Present Address: Department of Molecular and Cell Biology, University of California, Berkeley, Berkeley, CA 94720 USA

**Keywords:** Organelles, Nucleus, Size control, Intracellular scaling, Fission yeast, Nucleocytoplasmic transport, Nuclear envelope

## Abstract

Correlation between nuclear and cell size, the nucleocytoplasmic ratio, is a cellular phenomenon that has been reported throughout eukaryotes for more than a century but the mechanisms that achieve it are not well understood. Here, we review work that has shed light on the cellular processes involved in nuclear size control. These studies have implicated nucleocytoplasmic transport, LINC complexes, RNA processing, regulation of nuclear envelope expansion and partitioning of importin α in nuclear size control, moving us closer to a mechanistic understanding of this phenomenon.

## Nucleocytoplasmic ratio

“The constant, which we must accept as something given and not at present further analyzable, is the fixed proportion between nuclear volume and protoplasmic volume, namely, the karyoplasmic ratio.”

Theodor Boveri, 1905

More than a century ago, the study of sea urchin embryos led Hertwig to propose the Kern-Plasma-relation theory, suggesting that the ratio between nuclear size and cell size, the karyoplasmic or nucleocytoplasmic ratio, is a constant in all cell types (Hertwig 1903). Since then, a constant ratio between nuclear and cell size has been reported in many cell types from unicellular organisms such as the yeasts and Tetrahymena, to cells of multicellular animals and plants; in multicellular organisms, the nucleocytoplasmic ratio varies between cell types but is generally restricted to a narrow range for cells of a particular type (Boveri 1905; Conklin 1912; Edens et al. 2013; Gregory 2005; Hara and Kimura 2009; Jorgensen et al. 2007; Levy and Heald 2010; Neumann and Nurse 2007; Wilson 1925).

Maintenance of a constant nucleocytoplasmic ratio, although simply stated, is a complex regulatory process. It implies that cells are ‘aware’ of their overall size and that of their nucleus, and have a homeostatic mechanism (which could be passive or active in operation) coordinating nuclear and cell size. There must also be a mechanism operative that couples nuclear volume and nuclear membrane surface area growth as cells grow, even though these two parameters scale in different ways. This last point is because nuclear volume scales with *r*^3^ (where r is the radius of the nucleus, idealising it as a sphere) whilst nuclear surface area scales with *r*^2^. The molecular mechanisms underpinning this complex process are not well understood, but in recent years progress has been made in identifying the gene products that might be involved and in formulating various speculative models. These gene products and models are described in this review.

### Nucleoskeletal theory

Observation that nuclear size and cell size both usually correlate with cell ploidy (Gregory 2005; Wilson 1925) led to the proposal of the nucleoskeletal theory for determination of nuclear size (Cavalier-Smith 1982; Gregory 2005). In this model, it is proposed that DNA content determines nuclear size: the bulk DNA content of a cell sets a range for nuclear size, and within this range nuclear size is determined by the degree of compaction of DNA, which is regulated by specific proteins. The nuclear lamina and nuclear pore complexes were then proposed to associate with the appropriately folded DNA, with their level dependent on its size, and the lamina to recruit the phospholipid nuclear envelope (NE) giving rise to a nuclear size determined by DNA content (Cavalier-Smith 1982). The view that DNA content, and thus ploidy, directly determines nuclear size is probably the way most cell biologists think about nuclear size but this model does not provide an explanation for the experimental results described in the next section.

### Cell size, not DNA content, determines nuclear size

Several lines of evidence support the proposition that nuclear size is determined by cell size or a factor related to it, and not directly by DNA content. Nuclear volume correlates with cell volume across a range of cell sizes in both budding and fission yeasts (Jorgensen et al. 2007; Neumann and Nurse 2007). In fission yeast, genetic mutations and nutritional states were used to generate cells displaying a very wide 35-fold range of cell volumes. Nuclear volume correlated with cell volume across this range, maintaining a nuclear volume to cell volume (N/C) ratio of approximately 8% (Neumann and Nurse 2007). In pilot studies nuclear volume was found to increase steadily as cell volume increased during the cell cycle, maintaining a constant N/C ratio throughout interphase; there was no sudden increase in nuclear size accompanying DNA replication in S phase (Jorgensen et al. 2007; Neumann and Nurse 2007). Gradual nuclear growth as cells grow during the cell cycle was also observed in HeLa cells (Maeshima et al. 2011). These results suggested that DNA content did not directly determine nuclear volume. Furthermore, even a 16 fold increase in DNA content was insufficient to alter the N/C ratio of fission yeast cells that had undergone rereplication (Neumann and Nurse 2007).

Further evidence that nuclear size is causally dependent on cell size and not DNA content comes from results of nuclear transfer experiments. Transfer of the nucleus of a hen erythrocyte into the cytoplasm of a larger HeLa cell resulted in an increase in nuclear size (Harris 1967), as did transfer of HeLa cell nuclei into the cytoplasm of a larger *Xenopus* oocyte (Gurdon 1976). Nuclear size was also reported to increase concomitantly with cell size following treatment of murine hepatocytes with c–Myc (Kim et al. 2000), and to scale with cell size during the reductive divisions of post-16 cell stage *Caenorhabditis elegans* embryogenesis (Hara and Kimura 2009). In all of these situations, DNA content remained constant but nuclear size responded to changes in cell size.

These experiments and observations clearly establish that DNA content and ploidy do not determine nuclear size. So why is the nucleus larger in higher ploidy cells? The most likely explanation is that cells of higher ploidy undergo mitosis at an increased cell size (Jorgensen et al. 2007; Neumann and Nurse 2007), and it is this larger cell size that results in a larger nuclear size.

### Cytoplasmic factors influence nuclear volume

So how does cell size determine nuclear size? Useful contributions to our understanding have come from both in vivo studies and in vitro studies of reconstituted nuclei in cytoplasmic extracts. In centrifuged embryos of the sea snail *Crepidula plana*, nuclear size correlated with cytoplasmic volume and was not affected by changes of cell dimensions (Conklin 1912). In multinucleate *Schizosaccharomyces pombe* cells, nuclear volume correlated with the local cytoplasmic volume surrounding each nucleus; closely spaced nuclei surrounded by less cytoplasm grew at a slower rate and were, therefore, smaller than isolated nuclei surrounded by a larger cytoplasmic volume (Neumann and Nurse 2007). Similarly, in nuclear transfer experiments, when a cluster of HeLa cell nuclei was injected into the cytoplasm of a *Xenopus* oocyte, those on the edges of the cluster expanded faster than those closely packed in the middle (Gurdon 1976). These experiments suggest that there must be diffusible cytoplasmic factors that influence nuclear size.

Study of nuclear assembly in *Xenopus* egg extracts of two differentially sized species, the larger-celled pseudotetraploid *X. laevis* and the smaller-celled diploid *X. tropicalis*, has provided important insights (Levy and Heald 2010). The size of reconstituted nuclei was dependent on the species of origin of the cytoplasmic extract used rather than that of the DNA, supporting the idea that a diffusible cytoplasmic factor determines nuclear size. GFP-NLS accumulated at a faster rate in the larger nuclei derived from *X. laevis* egg extracts than those assembled in *X. tropicalis* extracts, leading the authors to propose that nucleocytoplasmic transport contributed to the difference in nuclear assembly rates in these in vitro extracts. A recent study demonstrated that importin α acts as a sensor of cell surface area to volume ratio, partitioning between the cytoplasm and the plasma membrane, regulating both mitotic spindle and nuclear size, and coordinating them with cell size, in *Xenopus* embryos and human cells (Brownlee and Heald 2019).

The fission yeast *S. pombe* is another useful system in which to study nuclear size control in vivo. *S. pombe* cells have a regular geometry facilitating calculation of cell volume (Mitchison 1957) and the nucleus is generally single copy and simply shaped so its volume can be approximated as a prolate spheroid (Neumann and Nurse 2007). It is a genetically tractable system and a gene deletion collection spanning 99% of *S. pombe* open reading frames is available facilitating near genome-wide systematic genetic screens.

Genetic screens for fission yeast mutants displaying aberrant N/C ratios have implicated a range of factors and biological processes in nuclear size control (Cantwell and Nurse 2019; Kume et al. 2017). A screen of *S. pombe* non-essential gene deletion mutants for those exhibiting aberrant N/C ratios identified mutants with high N/C ratios and a screen of *S. pombe* essential gene deletion mutants identified mutants with both high and low N/C ratios (Cantwell and Nurse 2019). These genome-wide screens have implicated the following processes in nuclear size control: bulk nucleocytoplasmic transport, transcription and RNA processing, LINC complexes and membrane expansion.

#### Bulk nucleocytoplasmic transport

It has been shown that perturbation of nucleocytoplasmic transport by treatment with leptomycin B, which inhibits the exportin CRM1 (Kudo et al. 1999), increases nuclear size in both *S. pombe* (Neumann and Nurse 2007) and mammalian cells (Ganguly et al. 2016). Two components of a complex implicated in nucleocytoplasmic transport were identified in the fission yeast N/C ratio screen (Kume et al. 2017). A third component of this complex, Rae1, is essential and a temperature sensitive *rae1*-*167* mutant when incubated at restrictive temperature generated enlarged nuclei. Nuclear protein accumulation was also observed in these cells concomitantly with the increase in N/C ratio and both phenotypes could be suppressed by inhibition of transcription or protein synthesis. More than 500 different proteins were found to accumulate in *rae1*-*167* nuclei at the restrictive temperature (Kume et al. 2017). The accumulated proteins were enriched for proteins reported to localise to the nucleus and subnuclear structures in wild type cells (Matsuyama et al. 2006) and were not mislocalised cytoplasmic proteins (Kume et al. 2017). It is possible that a few of these proteins specifically effect the nuclear size increase but we suggest that it is more likely that it is bulk accumulation of many proteins resulting from altered nucleocytoplasmic transport that changes the N/C ratio.

Perturbation of nucleocytoplasmic transport has been observed to influence N/C ratio and nuclear size in a range of systems, and mechanisms involving nuclear import of specific structural components of the nuclear envelope or lamina have also been proposed. Depletion of nucleoporin Nup188 in *Xenopus* egg extracts led to nuclear size increase due to increased trafficking of integral membrane proteins through the nuclear pore (Theerthagiri et al. 2010). Study of nuclear growth in *Xenopus* egg extracts of two differentially sized species indicated that the transport factors Impα2 and Ntf2 determine nuclear scaling in this system by regulating the import of lamin B3 (Levy and Heald 2010). The effect mediated by Ntf2 was later shown to be dependent on its interaction with Ran (Vukovic et al. 2016). Additionally, it was demonstrated that total lamin concentration, rather than the concentration of a specific lamin, influences nuclear size in *Xenopus* egg extracts and mammalian cells, and additionally increased lamin concentration has different effects at different developmental stages, sometimes increasing nuclear size and sometimes decreasing it (Jevtic et al. 2015). It was also reported that developmental changes in nuclear size may involve nuclear shrinking; lamin B3 phosphorylation by cPKC leads to its dissociation from the nuclear envelope and a reduction in nuclear size, leading to the proposal that the balance between nuclear expansion and shrinkage establishes nuclear size homeostasis (Edens et al. 2017; Edens and Levy 2014).

#### Transcription and RNA processing

Genes encoding proteins with functions in gene expression and RNA processing were enriched in genes deleted in N/C ratio mutants identified by visual screening in fission yeast (Cantwell and Nurse 2019). It is possible that perturbed RNA processing could lead to nuclear accumulation of defective mRNA transcripts and N/C ratio alteration by changes in bulk transport. Proteins with these functions are also often found as part of large complexes comprised of many protein and RNA components. Perturbing the stoichiometry or localisation of a large complex of this kind could disturb the nuclear pore and influence nucleocytoplasmic transport more generally, preventing the nucleocytoplasmic transport of other proteins, and thus leading to bulk transport effects on nuclear size.

#### LINC complexes

LINC complexes are conserved protein complexes that bridge the nuclear envelope, connecting nuclear chromatin to the cytoskeleton (Rothballer et al. 2013). In *S. pombe,* the KASH domain-containing integral outer nuclear membrane protein Kms2 and the SUN domain-containing integral inner nuclear membrane protein Sad1 form these bridging complexes (King et al. 2008). Both *kms2∆* and *sad1∆* were identified as N/C ratio mutant candidates (Cantwell and Nurse, 2019). Both mutants displayed enlarged N/C ratio phenotypes, suggesting that connection of chromatin to the cytoskeleton by LINC complexes may be important for N/C ratio control. It has been suggested that LINC complexes buffer forces on the nuclear envelope preserving nuclear morphology (King et al., 2008) and it is possible that they also act to constrain nuclear expansion contributing to nuclear size control. This proposal gets support from studies of mammalian cells which contain multiple KASH domain-containing proteins. Four of them are nesprins with a N-terminal actin-binding domain (ABD) separated from a C-terminal transmembrane KASH domain by an extended domain of spectrin repeats, and these are thought to form a filamentous network on the cytoplasmic face of the nuclear envelope (Lu et al. 2012). In HaCaT cells, disruption of these interchain interactions by overexpression of the ABD of Nesprin-2 increased nuclear size and expression of reduced length Nesprin-2 decreased nuclear size, demonstrating that the interactions between KASH domain proteins are important for nuclear size control in these cells.

#### Nuclear envelope expansion

Nuclear growth requires expansion of the nuclear envelope. Dysregulation of membrane synthesis in fission yeast by deletion of the gene encoding the catalytic, Nem1, or regulatory, Spo7, subunit of the phosphatase complex that regulates the lipin Ned1, led to overexpansion of the nuclear envelope and increased nuclear volume, indicating that accurate regulation of membrane expansion is important for nuclear size control (Kume et al. 2017). Combination of the *nem1∆* membrane synthesis mutant with the *rae1*-*167* nucleocytoplasmic transport mutant led to a greater N/C ratio than that observed in either single mutant, indicating that these are two distinct biological processes with roles in nuclear size control (Kume et al. 2017). The outer nuclear membrane is continuous with the endoplasmic reticulum (ER) and it has been proposed that nuclear envelope expansion is counteracted by conversion of ER membrane sheets to tubules by reticulon proteins. Overexpression of reticulon Rtn4 in U2OS cells was sufficient to limit nuclear expansion but its depletion was not sufficient to accelerate it (Anderson and Hetzer 2008).

Flow of membrane between the nuclear envelope and other membranous organelles also influences nuclear size. The inner nuclear membrane protein Lem2 has been shown to act as a barrier to membrane flow into and out of the nuclear envelope. Nuclei of fission yeast cells lacking Lem2 are more susceptible to size perturbation, undergoing rapid nuclear shrinkage when membrane synthesis is inhibited, suggesting that membrane flows out of the nuclear envelope. The endoplasmic reticulum protein Lnp1 is able to partially compensate for lack of Lem2 by buffering flow of membrane into and out of the nuclear envelope (Kume et al. 2019). These results have led to the proposal that the nucleocytoplasmic ratio may be maintained by overall membrane content within the cell scaling with overall cellular growth, combined with barrier proteins such as Lem2 and Lnp1 regulating membrane flow through the organellar membrane network. This flow could be regulated to ensure that a balance is maintained between the different membrane-bound organelles, bringing about a constant nucleocytoplasmic ratio.

### Why is nuclear size control important?

The constancy of the nucleocytoplasmic ratio in evolutionarily diverged yeast species (Jorgensen et al. 2007; Neumann and Nurse 2007) suggests that maintenance of a specific ratio is likely to be important. This could be because maintaining a constant N/C ratio enables cells to maintain coordination between transcription in the nucleoplasm and translation in the cytoplasm. Changes in N/C ratio have also been reported to be important for specific developmental transitions including the midblastula transition (MBT) of *Xenopus* embryogenesis (Jevtic and Levy 2015) and T cell activation (Gupta et al. 2012). Additionally, aberrant nuclear size and morphology are associated with disease (Smoyer and Jaspersen 2019; Zink et al. 2004), most notably cancer, suggesting that appropriate nuclear size control may be important for maintaining cell physiology; although whether nuclear size alteration is a contributor to, or downstream effect of, the disease pathologies remains to be determined.

## Conclusions

The studies discussed here have uncovered molecular players and biological processes involved in nuclear size control. These mechanistic insights provide us with ways to perturb nuclear size control and will facilitate studies of the physiological effects of aberrant nuclear size and its contribution to disease pathology. We now require a global understanding of how these processes are coordinated to bring about the nuclear size control that we observe. Whilst it is clear how some of the processes implicated could directly alter nuclear volume or surface area, for example Lem2 regulation of membrane flow into the nuclear envelope, for others it is less clear. How regulation of nuclear volume and surface area, two nuclear parameters that are inherently linked but exhibit different scaling relationships with respect to cell size, are integrated in nuclear size control remains enigmatic. Assessing the kinetics of recovery of nuclear size following perturbation will be important to address another open question in the field: is nuclear scaling a cellular property that is maintained passively as cells grow and divide or is it instead an actively controlled process? Hopefully, work in the range of eukaryotic systems described here over the coming years will see more progress in understanding nuclear size homeostasis, defined by Boveri in 1905.
